# CXCR4 antagonists for treatment of breast cancer

**DOI:** 10.18632/oncotarget.26090

**Published:** 2018-09-11

**Authors:** Javier Cortés, Esther Holgado, Jose Perez-Garcia

**Affiliations:** Javier Cortés: Ramon y Cajal University Hospital, Madrid, Spain; Vall d´Hebron Institute of Oncology, Barcelona, Spain; IOB Institute of Oncology, QuironGroup, Madrid & Barcelona, Spain

**Keywords:** CXCR4 antagonists, balixafortide, breast cancer, eribulin, oncology

Despite the introduction of new targeted therapies and cytotoxic agents, chemotherapy-pretreated metastatic breast cancer (MBC) remains an essentially incurable disease with only moderate median overall survival (OS) [1]. After failure of initial chemotherapy, there is no established standard of care for human epidermal growth factor receptor 2 (HER2)-negative women with MBC. Eribulin is licensed for MBC patients who have relapsed after anthracycline- and taxane-based chemotherapy. In a Phase 3 trial (EMBRACE), the median OS for eribulin or treatment of physician’s choice was 13.1 v 10.6 months, respectively [2]. There remains a need for more and better treatments that improve clinical outcomes and safety.

C-X-C chemokine receptor type 4 (CXCR4) is overexpressed in over 20 human tumor types. CXCR4 levels correlate with aggressive metastatic phenotypes and negative prognosis in breast cancer [1, 3]. Its natural ligand, stromal cell-derived factor-1α (SDF-1), is expressed highly at common sites of breast cancer metastases. The CXCR4/SDF-1 axis facilitates cancer cell survival by allowing tumors to migrate to SDF-1 rich sites, by evading the immune system, and by creating a favorable microenvironment for them to grow and metastasize; it might also protect breast tumors from cytotoxic chemotherapy [3-6]. Strategies that target the CXCR4/SDF-1axis could lead to promising cancer treatments.

Preclinical evidence suggests that disrupting CXCR4-dependent pathways might prevent the development of breast cancer metastases [1, 7]. Research has focused on the potential of CXCR4 antagonists to enhance the cytotoxic effect of chemotherapy and immunotherapy on tumors, and to regulate the tumor microenvironment and enhance endogenous anti-tumor responses by inhibiting T-regulatory cell-suppressive activity and increasing intratumoral penetration of CD8 T lymphocytes [4, 6]. Preclinical [8] and clinical [9] evidence suggests that CXCR4 antagonists may counteract the ability of tumor cells to evade the immune system by altering the distribution of immune cells and/or their activity in the tumor microenvironment. CXCR4 inhibition, *in vitro*, restores sensitivity to cytotoxic T-lymphocyte inhibitors and anti-programmed death-1 receptor ligand (anti-PDL-1) therapy; CXCR4 also appears to mediate resistance to endocrine therapies [1, 6].

CXCR4 antagonists are undergoing clinical trials for the treatment of various hematological and solid malignancies. In earlier published trials, efficacy was not always demonstrated at the doses investigated, and some CXCR4 antagonists have shown increased hematological risks when used alone or with chemotherapy [1].

Balixafortide is a potent, selective CXCR4 antagonist. In murine models of triple-negative breast cancer (TNBC), POL5551 (a balixafortide analogue) + eribulin showed enhanced cytotoxic activity and inhibition of metastases compared with eribulin alone [10].

Important data were published recently from the first Phase 1 trial to assess eribulin + balixafortide in HER2-negative, CXCR4-positive women who had previously received 1−3 chemotherapy regimens for MBC [1]. In this single-arm, dose escalation trial, patients received eribulin with increasing doses of balixafortide (0∙5−5.5mg/kg) using a 3+3 design. In total, 56 women were enrolled and treated; as no dose limiting toxicities were observed during dose escalation, the cohort on the highest balixafortide dose (5∙5mg/kg) was expanded to 25 patients to confirm safety and anti-tumor activity.

Patients in the Expanded Cohort (EC) had an objective response rate (ORR) of 38% and a clinical benefit rate (CBR) of 63%. In the Overall Efficacy Population (OEP; all doses of balixafortide), the ORR was 30% and the CBR was 44%. One-year OS was 75% in the EC (62% in the OEP) and median progression free survival (PFS) was 6∙2 months in the EC (4∙6 months in the OEP).

Although inter-trial comparisons should be interpreted with caution, these response rates, especially for the EC, are much higher than those reported for eribulin monotherapy in similar MBC populations (ORR 9−12%, CBR 17%), and the OS and PFS are also higher than reported for eribulin monotherapy (one year OS 54%, median PFS 2∙6–4∙1 months) [1].

While the trial was small, the sample size for the EC was similar to those in other translational studies and provides a reasonable precision of the anti-tumor effect. Twenty-three percent of patients had TNBC.

Not adding to the burden of side-effects is an important factor when considering further treatment in heavily pre-treated patients with MBC. The safety and tolerability of balixafortide + eribulin appeared comparable to published data on either eribulin or balixafortide monotherapy [1]. Even at the highest balixafortide dose (5.5mg/kg), the frequency of most treatment-emergent adverse events (including Grade 3/4 neutropenia, leukopenia or peripheral neuropathy) was similar to that reported in other studies for eribulin monotherapy.

The promising results of this combination might be further enhanced, and other combinations with CXCR4 antagonists (e.g. with immune checkpoint inhibitors) are also worthy of investigation.

It is not yet known whether CXCR4 antagonists will improve the outcome of pretreated MBC. However, the highly encouraging data observed with balixafortide and eribulin have opened an exciting opportunity for patients that will be explored in future randomized trials.
